# Computational neuroscience in research for depression

**DOI:** 10.1186/2193-9616-1-19

**Published:** 2013-12-19

**Authors:** Georg Juckel

**Affiliations:** Department of Psychiatry, Psychotherapy and Preventive Medicine Ruhr University Bochum, Alexandrinenstr 1-3, 44791 Bochum, Germany

**Keywords:** Depression, Computational neuroscience, Serotonin, Mathematical modeling

## Abstract

Depression is a common and hazardous mental disorder, which has been pathophysiologically associated with alterations of neurocircuitries involving medial prefrontal cortex, hippocampus and thalamus. Recent progress in computational neuroscience, particularly in the field of in silico psychopharmacology suggests the increasing potential of mathematical modeling in providing insights on the dynamics of these neuronal networks, which in turn may lead to further develop and clarify the present models of the pathophysiology of depression. Moreover, computational approaches provide well-defined non-invasive frameworks for investigation of the clinically common poly-pharmacological treatment strategies, which take us one step closer to the development of novel agents that will potentially result in diagnostic and prognostic indicators to be used in individualized treatment strategies.

## Purpose

Research of psychiatric diseases such as depression is complicated thereby, that direct measurements are not possible and validation models are available on a limited basis.

## Main text

For the last two decades the prevalence of depression has experienced an increase worldwide, starting with 10% at the end of the 80’s and is now counting 25% (Wittchen et al. 2011). These are only partly absolute increases because of the permanent rise of pressure to perform not only professionally but also privately. But there is also a relative increase since in the past, many affected people had received a different–more somatic–diagnosis due to stigmatizations in classification of psychiatric disorders. Depression is one of the most common and hazardous diseases in the human population. Symptoms of physical, mental and emotional ailment are considered to be dramatic for both the affected person as well as for the family. Furthermore, depression is potentially lethal because of the considerably high suicide rates of above 10%, the estimatededly high number of unreported cases of suicide and continuously prevailing agonizing, suicidal thoughts and stimuli. In addition, the last two decades revealed that depression is a longitudinal disease, encasing one’s life story and development dynamically. We have to assume that genetic and neurologic factors at embryonic age and early childhood associated with critical life events (like death, violence, separation, parting experiences, trauma) trigger a mental condition in the affected person, that lead to an outbreak of the depressive disease when stress increases in the adult (e.g. at work or due to private challenges) or when neurobiological changes occur (e.g. during menopausal estrogen reduction in females). Once appeared, depressive states frequently recur in a lifetime.

However, most pivotal for these diseases is certainly the neuronal correlate. Emanating from the monamine hypothesis of the 60’s postulating a serotonin and noradrenalin deficit in depressive patients, in the 70’s and 80’s perceptions evolved implying a change in the hypothalamic–pituitary–adrenal axis. Findings of hypercortisolism in many (but not all) patients attested to this claim. Many pharmacologic drugs were developed but couldn’t be used due to severe medical side effects.

In the last years, the concept of depression representing a dysregulation in brain circuitries has been empirically secured. In depression the brain is affected ubiquitary. There is no global change of specific transmitters but there seem to be dysregulating phenomena in certain brain circuits between thalamus, hippocampus, amygdala, parts of the prefrontal cortex as well as other brain regions, which are of microstructured nature and can be found for example in neuron populations such as the GABAergic interneurons. The cause of these dysregulation is however still mysterious. We assume that, apart from the hippocampus, especially the medial prefrontal cortex plays a key role in the pathophysiology of depression (Juckel et al. 1999; Klein et al. 2010). These partly multifunctional changes as well as the projection fibers are not illustratable with the present imaging techniques such as structural magnetic resonance imaging (MRI), functional magnetic resonance imaging (fMRI), and positron emission tomography (PET). Moreover, the present technologies do not allow an adequate examination of the multi-scale interactions of the alterations at neurocircuitry level with for instance genetics of 5-HT transporters.

## Discussion

Here we have to acknowledge clearly the domain of the newly developing computational neuroscience, i.e. the mathematical modeling of brain circuitries and their changes. It is through mathematical modeling of neuronal networks, that we are able to monitor and alter functional and dysfunctional states under systematic and standardized conditions and that important conclusions can be drawn upon the conditional structure of modified brain circuits, e.g. in depression. In fact, it is imaginable that computational neuroscience will lead to narrowing down the question about the causative source depending upon the integration of assumption of models relating to one or two brain regions in order to examine them later using human or animal experiments. This would include the possibility of examining the operating mechanisms and mightiness of the few more locally/regionally active somato therapies in the treatment of depression, such as deep brain stimulation, transcranial magnetic stimulation or-with limitations-the EKT.

Furthermore, it is of vital importance to apply mathematical models to investigate the action of psychiatric drugs. In the clinical routine we use antidepressants of the SSRI type as a mono-therapy. We are insufficiently informed about the regulatory characteristics of serotonergic neurons in neuronal feedback cycles and the glutamatergic projection fibers (of pyramidal cells) acting with GABAergic interneurons. Furthermore, the impact of the blockade of serotonin transporters particularly on the somatodendritic autoreceptors 5-HT_1A_ but also of the terminal 5-HT_1B_ receptors is still not clear (Jacobs and Azmitia, 1992), which could be investigated by computational approaches. Modeling such cascades would greatly contribute to understanding not only the pathophysiology of depression but also to comprehending what operating mechanism lead to improvement of depression by antidepressants. Moreover, we can recognize the potential of applying computational neuroscience to psychopharmacology to optimize the treatment of depression particularly with regard to the prevalence of poly-pharmacological treatment strategies. For instance, we know little about the interactions that are taking place directly in the brain between antidepressants and benzodiazepines or mood stabilizers such as lithium and valproate. By modeling drug interaction interactions it is possible to draw several conclusions from a specific anatomic pathophysiological understanding with respect to the mode of action as well as the cause of side effects.

## Conclusions

In summary, in terms of psychiatry and depression in particular, computational neuroscience represents a very important column in future research and the entire field of psychiatry and psychotherapy, especially biological psychiatry would benefit from its outcomes.

Computational neuroscience describes a very plausible and future-oriented non-invasive attempt to examine and describe neuronal processes and their modifications in psychiatric diseases like depression, psychoactive substances and psychotherapeutic strategies.
